# Supplementary material for: UBVRI night sky brightness at Kottamia Astronomical Observatory
Source: Sci Rep. 2023 Oct 5;13:16754. doi: 10.1038/s41598-023-43844-x (PMC10556021; doi:10.1038/s41598-023-43844-x)
Supplement: Supplementary file 1 — Supplementary Information. [file 41598_2023_43844_MOESM1_ESM.docx]

**Supplementary Material for UBVRI night sky brightness at Kottamia Astronomical Observatory**

**Mohamed F. Aboushelib^1 ✉^, A.B. Morcos^1^, S. Nawar^1^, Shalabiea, O. M.^2,3^ and Awad, Z.^2^**

^1^National Research Institute of Astronomy and Geophysics, Helwan, Cairo, Egypt

^2^Astronomy, Space Science and Meteorology Department, Faculty of Science, Cairo University, Giza, Egypt

^3^Faculty of Navigation Science and Space Technology, Beni-Suef University, Beni-Suef, Egypt

**Contents of This File**

- Introduction
- Tables S-1 through S-10
- Figures S-1 through S-4

**Introduction**

Tables from S-1 to S-10 show the average NSB in different colors (filters), and the color indices over the three observing nights at different Az degrees and Alt from 0^o^ to 90°. Figures from S-1 to S-4 show the variation of the average sky brightness in the UBVRI filters and the standard errors with different azimuth degrees at altitude 0°, 30°, 60° and 90°, respectively.

**Table S-1** The average NSB in different colors, and the color indices over the three observing nights at Alt 0^o^

| Az  Degrees | Alt  Degrees | U_avg_  mag/arcsec^2^ | B_avg_  mag/arcsec^2^ | V_avg_  mag/arcsec^2^ | R_avg_  mag/arcsec^2^ | I_avg_  mag/arcsec^2^ |  | U-B | B-V | V-R | R-I |
| --- | --- | --- | --- | --- | --- | --- | --- | --- | --- | --- | --- |
|  |  |  |  |  |  |  |  |  |  |  |  |
| 0 | 0 | 19.07 | 19.43 | 17.11 | 16.98 | 16.67 |  | -0.37 | 2.32 | 0.13 | 0.31 |
| 10 | 0 | 20.20 | 19.47 | 18.34 | 17.62 | 17.56 |  | 0.73 | 1.13 | 0.72 | 0.06 |
| 20 | 0 | 21.32 | 20.91 | 19.97 | 19.13 | 18.68 |  | 0.40 | 0.94 | 0.85 | 0.44 |
| 30 | 0 | 21.04 | 21.17 | 19.88 | 18.86 | 18.42 |  | -0.14 | 1.30 | 1.01 | 0.44 |
| 40 | 0 | 20.19 | 20.31 | 19.09 | 18.24 | 17.74 |  | -0.12 | 1.22 | 0.85 | 0.50 |
| 50 | 0 | 20.33 | 20.83 | 19.51 | 18.66 | 18.09 |  | -0.50 | 1.32 | 0.84 | 0.57 |
| 60 | 0 | 20.53 | 20.62 | 19.46 | 18.62 | 17.98 |  | -0.09 | 1.16 | 0.84 | 0.64 |
| 70 | 0 | 20.32 | 20.63 | 19.38 | 18.54 | 17.84 |  | -0.31 | 1.25 | 0.84 | 0.70 |
| 80 | 0 | 20.16 | 20.50 | 19.11 | 18.24 | 17.69 |  | -0.34 | 1.39 | 0.87 | 0.55 |
| 90 | 0 | 20.41 | 20.92 | 19.37 | 18.45 | 17.89 |  | -0.51 | 1.55 | 0.91 | 0.56 |
| 100 | 0 | 20.00 | 20.39 | 18.21 | 17.04 | 16.69 |  | -0.40 | 2.19 | 1.17 | 0.36 |
| 110 | 0 | 20.39 | 20.70 | 19.45 | 18.59 | 17.89 |  | -0.31 | 1.25 | 0.86 | 0.71 |
| 120 | 0 | 20.39 | 20.69 | 19.34 | 18.50 | 17.82 |  | -0.30 | 1.35 | 0.85 | 0.68 |
| 130 | 0 | 19.78 | 19.95 | 18.90 | 18.03 | 17.49 |  | -0.17 | 1.05 | 0.87 | 0.54 |
| 140 | 0 | 19.94 | 20.28 | 18.80 | 17.89 | 17.03 |  | -0.34 | 1.48 | 0.91 | 0.87 |
| 150 | 0 | 20.10 | 20.36 | 19.08 | 18.22 | 17.31 |  | -0.27 | 1.28 | 0.85 | 0.91 |
| 160 | 0 | 20.29 | 20.32 | 19.08 | 18.27 | 17.41 |  | -0.03 | 1.24 | 0.81 | 0.86 |
| 170 | 0 | 20.48 | 20.61 | 19.31 | 18.47 | 17.80 |  | -0.13 | 1.30 | 0.84 | 0.67 |
| 180 | 0 | 20.70 | 21.08 | 19.62 | 18.79 | 18.10 |  | -0.38 | 1.46 | 0.83 | 0.69 |
| 190 | 0 | 20.76 | 21.06 | 19.63 | 18.85 | 18.01 |  | -0.30 | 1.44 | 0.77 | 0.84 |
| 200 | 0 | 20.82 | 21.16 | 19.75 | 18.93 | 18.13 |  | -0.34 | 1.41 | 0.82 | 0.80 |
| 210 | 0 | 20.85 | 21.23 | 19.81 | 18.98 | 18.26 |  | -0.38 | 1.41 | 0.83 | 0.73 |
| 220 | 0 | 20.70 | 20.97 | 19.67 | 18.84 | 18.16 |  | -0.27 | 1.30 | 0.83 | 0.67 |
| 230 | 0 | 20.29 | 20.73 | 19.23 | 18.41 | 17.83 |  | -0.44 | 1.50 | 0.81 | 0.58 |
| 240 | 0 | 19.85 | 19.99 | 18.60 | 17.81 | 17.33 |  | -0.15 | 1.40 | 0.79 | 0.48 |
| 250 | 0 | 17.22 | 17.10 | 15.58 | 14.80 | 14.65 |  | 0.11 | 1.52 | 0.78 | 0.15 |
| 260 | 0 | 19.20 | 19.14 | 17.69 | 16.89 | 16.56 |  | 0.06 | 1.45 | 0.80 | 0.33 |
| 270 | 0 | 19.36 | 19.17 | 17.85 | 17.02 | 16.73 |  | 0.19 | 1.33 | 0.82 | 0.29 |
| 280 | 0 | 18.71 | 18.55 | 17.26 | 16.45 | 16.20 |  | 0.16 | 1.30 | 0.81 | 0.24 |
| 290 | 0 | 18.72 | 18.40 | 17.27 | 16.53 | 16.13 |  | 0.31 | 1.13 | 0.74 | 0.39 |
| 300 | 0 | 18.33 | 17.85 | 16.61 | 15.80 | 15.57 |  | 0.48 | 1.24 | 0.81 | 0.23 |
| 310 | 0 | 18.40 | 17.93 | 16.68 | 15.88 | 15.64 |  | 0.47 | 1.25 | 0.79 | 0.25 |
| 320 | 0 | 17.51 | 16.79 | 15.62 | 14.85 | 14.46 |  | 0.72 | 1.17 | 0.77 | 0.39 |
| 330 | 0 | 17.93 | 17.35 | 16.07 | 15.33 | 15.17 |  | 0.58 | 1.27 | 0.74 | 0.17 |
| 340 | 0 | 17.96 | 17.05 | 15.87 | 15.20 | 14.80 |  | 0.91 | 1.18 | 0.67 | 0.40 |
| 350 | 0 | 18.93 | 18.78 | 17.23 | 16.50 | 16.23 |  | 0.15 | 1.55 | 0.73 | 0.26 |
| 360 | 0 | 19.12 | 19.41 | 17.77 | 17.03 | 16.65 |  | -0.29 | 1.64 | 0.75 | 0.38 |

**Table S-2** The average NSB in different colors and the color indices over the three observing nights at Alt 10^o^

| Az  Degrees | Alt  Degrees | U_avg_  mag/arcsec^2^ | B_avg_  mag/arcsec^2^ | V_avg_  mag/arcsec^2^ | R_avg_  mag/arcsec^2^ | I_avg_  mag/arcsec^2^ |  | U-B | B-V | V-R | R-I |
| --- | --- | --- | --- | --- | --- | --- | --- | --- | --- | --- | --- |
|  |  |  |  |  |  |  |  |  |  |  |  |
| 0 | 10 | 18.87 | 19.14 | 17.82 | 17.05 | 16.68 |  | -0.27 | 1.32 | 0.77 | 0.37 |
| 10 | 10 | 20.14 | 19.44 | 18.28 | 17.59 | 17.55 |  | 0.70 | 1.15 | 0.70 | 0.03 |
| 20 | 10 | 20.25 | 19.80 | 18.44 | 17.77 | 17.69 |  | 0.45 | 1.36 | 0.67 | 0.07 |
| 30 | 10 | 21.04 | 20.86 | 19.43 | 18.73 | 18.37 |  | 0.19 | 1.42 | 0.70 | 0.36 |
| 40 | 10 | 19.89 | 20.06 | 18.77 | 18.04 | 17.43 |  | -0.17 | 1.29 | 0.74 | 0.61 |
| 50 | 10 | 20.09 | 20.21 | 18.97 | 18.23 | 17.57 |  | -0.13 | 1.24 | 0.74 | 0.66 |
| 60 | 10 | 20.15 | 20.21 | 19.08 | 18.34 | 17.63 |  | -0.06 | 1.13 | 0.74 | 0.71 |
| 70 | 10 | 20.12 | 20.29 | 19.13 | 18.36 | 17.63 |  | -0.17 | 1.16 | 0.77 | 0.73 |
| 80 | 10 | 20.09 | 20.31 | 19.13 | 18.36 | 17.64 |  | -0.23 | 1.19 | 0.77 | 0.72 |
| 90 | 10 | 20.09 | 20.30 | 19.12 | 18.34 | 17.64 |  | -0.21 | 1.18 | 0.78 | 0.70 |
| 100 | 10 | 20.15 | 20.33 | 19.15 | 18.37 | 17.69 |  | -0.18 | 1.17 | 0.78 | 0.68 |
| 110 | 10 | 20.15 | 20.31 | 19.21 | 18.39 | 17.72 |  | -0.16 | 1.11 | 0.82 | 0.67 |
| 120 | 10 | 20.07 | 20.26 | 19.13 | 18.29 | 17.65 |  | -0.20 | 1.13 | 0.84 | 0.63 |
| 130 | 10 | 20.05 | 20.28 | 19.13 | 18.29 | 17.65 |  | -0.22 | 1.15 | 0.83 | 0.65 |
| 140 | 10 | 20.10 | 20.32 | 19.22 | 18.40 | 17.68 |  | -0.22 | 1.10 | 0.82 | 0.72 |
| 150 | 10 | 20.31 | 20.34 | 19.32 | 18.51 | 17.76 |  | -0.03 | 1.02 | 0.80 | 0.76 |
| 160 | 10 | 20.34 | 20.40 | 19.34 | 18.56 | 17.71 |  | -0.06 | 1.06 | 0.78 | 0.86 |
| 170 | 10 | 20.41 | 20.45 | 19.37 | 18.61 | 17.75 |  | -0.04 | 1.08 | 0.75 | 0.86 |
| 180 | 10 | 20.29 | 20.37 | 19.32 | 18.56 | 17.68 |  | -0.08 | 1.05 | 0.75 | 0.88 |
| 190 | 10 | 20.21 | 20.25 | 19.19 | 18.46 | 17.49 |  | -0.03 | 1.06 | 0.74 | 0.97 |
| 200 | 10 | 20.09 | 20.21 | 19.12 | 18.35 | 17.52 |  | -0.12 | 1.08 | 0.77 | 0.84 |
| 210 | 10 | 19.89 | 20.08 | 18.95 | 18.19 | 17.50 |  | -0.19 | 1.13 | 0.76 | 0.69 |
| 220 | 10 | 19.61 | 19.86 | 18.71 | 17.94 | 17.33 |  | -0.25 | 1.15 | 0.76 | 0.61 |
| 230 | 10 | 19.30 | 19.59 | 18.42 | 17.65 | 17.09 |  | -0.29 | 1.17 | 0.77 | 0.55 |
| 240 | 10 | 18.99 | 19.29 | 18.07 | 17.35 | 16.84 |  | -0.29 | 1.22 | 0.72 | 0.50 |
| 250 | 10 | 18.66 | 19.01 | 17.76 | 17.02 | 16.56 |  | -0.35 | 1.25 | 0.74 | 0.46 |
| 260 | 10 | 18.51 | 18.85 | 17.58 | 16.81 | 16.41 |  | -0.34 | 1.27 | 0.77 | 0.40 |
| 270 | 10 | 18.31 | 18.65 | 17.38 | 16.58 | 16.21 |  | -0.34 | 1.27 | 0.80 | 0.37 |
| 280 | 10 | 18.09 | 18.41 | 17.16 | 16.33 | 15.98 |  | -0.32 | 1.25 | 0.83 | 0.35 |
| 290 | 10 | 17.90 | 18.29 | 17.00 | 16.17 | 15.82 |  | -0.39 | 1.29 | 0.84 | 0.35 |
| 300 | 10 | 17.87 | 18.26 | 16.97 | 16.15 | 15.82 |  | -0.40 | 1.29 | 0.82 | 0.33 |
| 310 | 10 | 17.78 | 18.24 | 16.92 | 16.11 | 15.78 |  | -0.45 | 1.32 | 0.81 | 0.33 |
| 320 | 10 | 17.57 | 18.15 | 16.74 | 15.94 | 15.63 |  | -0.58 | 1.41 | 0.80 | 0.31 |
| 330 | 10 | 17.74 | 18.33 | 16.89 | 16.10 | 15.78 |  | -0.59 | 1.43 | 0.79 | 0.32 |
| 340 | 10 | 18.02 | 18.52 | 17.11 | 16.33 | 15.98 |  | -0.49 | 1.40 | 0.79 | 0.35 |
| 350 | 10 | 18.26 | 18.74 | 17.33 | 16.53 | 16.17 |  | -0.48 | 1.41 | 0.80 | 0.37 |
| 360 | 10 | 18.91 | 19.16 | 17.90 | 17.08 | 16.72 |  | -0.25 | 1.26 | 0.82 | 0.36 |

**Table S-3** The average NSB in different colors and the color indices over the three observing nights at Alt 20^o^

| Az  Degrees | Alt  Degrees | U_avg_  mag/arcsec^2^ | B_avg_  mag/arcsec^2^ | V_avg_  mag/arcsec^2^ | R_avg_  mag/arcsec^2^ | I_avg_  mag/arcsec^2^ |  | U-B | B-V | V-R | R-I |
| --- | --- | --- | --- | --- | --- | --- | --- | --- | --- | --- | --- |
|  |  |  |  |  |  |  |  |  |  |  |  |
| 0 | 20 | 19.17 | 19.28 | 18.16 | 17.38 | 16.99 |  | -0.11 | 1.13 | 0.78 | 0.39 |
| 10 | 20 | 19.44 | 19.53 | 18.41 | 17.64 | 17.18 |  | -0.09 | 1.12 | 0.77 | 0.45 |
| 20 | 20 | 19.69 | 19.72 | 18.62 | 17.86 | 17.34 |  | -0.03 | 1.09 | 0.76 | 0.51 |
| 30 | 20 | 19.88 | 19.89 | 18.81 | 18.05 | 17.48 |  | -0.01 | 1.08 | 0.77 | 0.56 |
| 40 | 20 | 20.01 | 20.02 | 18.98 | 18.22 | 17.59 |  | -0.01 | 1.04 | 0.76 | 0.63 |
| 50 | 20 | 20.15 | 20.14 | 19.11 | 18.35 | 17.70 |  | 0.01 | 1.03 | 0.76 | 0.65 |
| 60 | 20 | 20.24 | 20.22 | 19.15 | 18.39 | 17.68 |  | 0.01 | 1.08 | 0.75 | 0.71 |
| 70 | 20 | 20.16 | 20.24 | 19.21 | 18.47 | 17.75 |  | -0.07 | 1.03 | 0.73 | 0.72 |
| 80 | 20 | 20.18 | 20.31 | 19.24 | 18.51 | 17.76 |  | -0.13 | 1.07 | 0.74 | 0.74 |
| 90 | 20 | 20.21 | 20.34 | 19.26 | 18.51 | 17.79 |  | -0.13 | 1.08 | 0.75 | 0.72 |
| 100 | 20 | 20.26 | 20.37 | 19.27 | 18.51 | 17.82 |  | -0.12 | 1.11 | 0.75 | 0.70 |
| 110 | 20 | 20.24 | 20.41 | 19.28 | 18.53 | 17.84 |  | -0.17 | 1.12 | 0.76 | 0.69 |
| 120 | 20 | 20.25 | 20.42 | 19.26 | 18.51 | 17.80 |  | -0.17 | 1.16 | 0.75 | 0.71 |
| 130 | 20 | 20.27 | 20.47 | 19.28 | 18.54 | 17.82 |  | -0.21 | 1.19 | 0.74 | 0.72 |
| 140 | 20 | 20.28 | 20.48 | 19.31 | 18.58 | 17.84 |  | -0.20 | 1.17 | 0.73 | 0.74 |
| 150 | 20 | 20.30 | 20.47 | 19.31 | 18.59 | 17.81 |  | -0.16 | 1.15 | 0.73 | 0.78 |
| 160 | 20 | 20.25 | 20.40 | 19.26 | 18.52 | 17.64 |  | -0.15 | 1.14 | 0.74 | 0.88 |
| 170 | 20 | 20.25 | 20.34 | 19.25 | 18.52 | 17.63 |  | -0.09 | 1.09 | 0.73 | 0.89 |
| 180 | 20 | 20.27 | 19.79 | 19.19 | 18.51 | 17.49 |  | 0.48 | 0.59 | 0.68 | 1.02 |
| 190 | 20 | 20.17 | 20.12 | 19.13 | 18.44 | 17.50 |  | 0.05 | 0.98 | 0.69 | 0.94 |
| 200 | 20 | 20.09 | 20.10 | 19.07 | 18.38 | 17.57 |  | 0.00 | 1.02 | 0.69 | 0.81 |
| 210 | 20 | 19.95 | 20.02 | 18.91 | 18.22 | 17.51 |  | -0.08 | 1.11 | 0.69 | 0.71 |
| 220 | 20 | 19.78 | 19.88 | 18.77 | 18.09 | 17.45 |  | -0.10 | 1.12 | 0.68 | 0.64 |
| 230 | 20 | 19.56 | 19.70 | 18.59 | 17.90 | 17.32 |  | -0.15 | 1.11 | 0.69 | 0.58 |
| 240 | 20 | 19.33 | 19.55 | 18.38 | 17.67 | 17.15 |  | -0.21 | 1.17 | 0.71 | 0.51 |
| 250 | 20 | 19.14 | 19.36 | 18.16 | 17.46 | 16.99 |  | -0.22 | 1.20 | 0.71 | 0.47 |
| 260 | 20 | 18.95 | 19.19 | 17.98 | 17.26 | 16.81 |  | -0.24 | 1.21 | 0.72 | 0.45 |
| 270 | 20 | 18.78 | 19.04 | 17.81 | 17.08 | 16.68 |  | -0.26 | 1.23 | 0.73 | 0.40 |
| 280 | 20 | 18.61 | 18.89 | 17.65 | 16.91 | 16.53 |  | -0.28 | 1.24 | 0.74 | 0.38 |
| 290 | 20 | 18.49 | 18.77 | 17.53 | 16.79 | 16.43 |  | -0.27 | 1.24 | 0.74 | 0.35 |
| 300 | 20 | 18.42 | 18.72 | 17.46 | 16.71 | 16.37 |  | -0.30 | 1.26 | 0.75 | 0.34 |
| 310 | 20 | 18.35 | 18.68 | 17.40 | 16.64 | 16.32 |  | -0.33 | 1.28 | 0.76 | 0.32 |
| 320 | 20 | 18.23 | 18.65 | 17.30 | 16.53 | 16.26 |  | -0.41 | 1.34 | 0.77 | 0.27 |
| 330 | 20 | 18.39 | 18.75 | 17.44 | 16.68 | 16.37 |  | -0.36 | 1.32 | 0.76 | 0.31 |
| 340 | 20 | 18.60 | 18.95 | 17.63 | 16.88 | 16.53 |  | -0.35 | 1.32 | 0.75 | 0.35 |
| 350 | 20 | 18.86 | 19.12 | 17.84 | 17.10 | 16.65 |  | -0.27 | 1.28 | 0.74 | 0.44 |
| 360 | 20 | 19.19 | 19.33 | 18.16 | 17.42 | 16.97 |  | -0.14 | 1.17 | 0.74 | 0.45 |

**Table S-4** The average NSB in different colors and the color indices over the three observing nights at Alt 30^o^

| Az  Degrees | Alt  Degrees | U_avg_  mag/arcsec^2^ | B_avg_  mag/arcsec^2^ | V_avg_  mag/arcsec^2^ | R_avg_  mag/arcsec^2^ | I_avg_  mag/arcsec^2^ |  | U-B | B-V | V-R | R-I |
| --- | --- | --- | --- | --- | --- | --- | --- | --- | --- | --- | --- |
|  |  |  |  |  |  |  |  |  |  |  |  |
| 0 | 30 | 19.53 | 19.58 | 18.50 | 17.77 | 17.28 |  | -0.05 | 1.07 | 0.73 | 0.50 |
| 10 | 30 | 19.70 | 19.80 | 18.68 | 17.96 | 17.41 |  | -0.10 | 1.12 | 0.72 | 0.55 |
| 20 | 30 | 19.83 | 19.94 | 18.84 | 18.12 | 17.54 |  | -0.11 | 1.10 | 0.72 | 0.58 |
| 30 | 30 | 20.04 | 20.06 | 19.00 | 18.27 | 17.66 |  | -0.02 | 1.07 | 0.72 | 0.62 |
| 40 | 30 | 20.16 | 20.17 | 19.10 | 18.40 | 17.74 |  | -0.01 | 1.07 | 0.70 | 0.66 |
| 50 | 30 | 20.23 | 20.27 | 19.20 | 18.48 | 17.80 |  | -0.04 | 1.07 | 0.72 | 0.68 |
| 60 | 30 | 20.22 | 20.37 | 19.24 | 18.53 | 17.79 |  | -0.16 | 1.13 | 0.71 | 0.73 |
| 70 | 30 | 20.37 | 20.36 | 19.34 | 18.63 | 17.89 |  | 0.01 | 1.02 | 0.71 | 0.74 |
| 80 | 30 | 20.41 | 20.41 | 19.39 | 18.68 | 17.93 |  | -0.01 | 1.02 | 0.71 | 0.74 |
| 90 | 30 | 20.43 | 20.48 | 19.41 | 18.70 | 17.95 |  | -0.05 | 1.07 | 0.71 | 0.75 |
| 100 | 30 | 20.43 | 20.48 | 19.38 | 18.66 | 17.93 |  | -0.05 | 1.11 | 0.72 | 0.73 |
| 110 | 30 | 20.35 | 20.48 | 19.36 | 18.65 | 17.90 |  | -0.13 | 1.12 | 0.71 | 0.75 |
| 120 | 30 | 20.36 | 20.49 | 19.38 | 18.65 | 17.93 |  | -0.13 | 1.12 | 0.73 | 0.72 |
| 130 | 30 | 20.37 | 20.50 | 19.38 | 18.63 | 17.94 |  | -0.13 | 1.12 | 0.76 | 0.69 |
| 140 | 30 | 20.26 | 20.49 | 19.35 | 18.60 | 17.91 |  | -0.24 | 1.14 | 0.75 | 0.70 |
| 150 | 30 | 20.37 | 20.46 | 19.42 | 18.70 | 17.97 |  | -0.10 | 1.04 | 0.73 | 0.72 |
| 160 | 30 | 20.37 | 20.37 | 19.39 | 18.65 | 17.89 |  | 0.00 | 0.98 | 0.74 | 0.77 |
| 170 | 30 | 20.36 | 20.32 | 19.36 | 18.63 | 17.79 |  | 0.04 | 0.96 | 0.74 | 0.83 |
| 180 | 30 | 20.22 | 20.37 | 19.27 | 18.53 | 17.79 |  | -0.15 | 1.09 | 0.75 | 0.74 |
| 190 | 30 | 20.29 | 20.26 | 19.27 | 18.51 | 17.85 |  | 0.03 | 0.99 | 0.76 | 0.66 |
| 200 | 30 | 20.15 | 20.21 | 19.19 | 18.44 | 17.81 |  | -0.06 | 1.02 | 0.75 | 0.64 |
| 210 | 30 | 20.08 | 20.16 | 19.09 | 18.34 | 17.73 |  | -0.08 | 1.07 | 0.75 | 0.61 |
| 220 | 30 | 19.98 | 20.07 | 18.99 | 18.25 | 17.68 |  | -0.09 | 1.08 | 0.74 | 0.57 |
| 230 | 30 | 19.81 | 19.93 | 18.83 | 18.09 | 17.57 |  | -0.13 | 1.10 | 0.74 | 0.52 |
| 240 | 30 | 19.66 | 19.77 | 18.69 | 17.94 | 17.46 |  | -0.11 | 1.08 | 0.75 | 0.48 |
| 250 | 30 | 19.51 | 19.62 | 18.55 | 17.79 | 17.35 |  | -0.11 | 1.07 | 0.76 | 0.43 |
| 260 | 30 | 19.35 | 19.48 | 18.39 | 17.62 | 17.20 |  | -0.14 | 1.10 | 0.77 | 0.42 |
| 270 | 30 | 19.23 | 19.37 | 18.27 | 17.49 | 17.07 |  | -0.14 | 1.11 | 0.77 | 0.42 |
| 280 | 30 | 19.11 | 19.22 | 18.15 | 17.37 | 17.00 |  | -0.12 | 1.08 | 0.78 | 0.37 |
| 290 | 30 | 19.01 | 19.22 | 18.05 | 17.27 | 16.92 |  | -0.21 | 1.16 | 0.78 | 0.35 |
| 300 | 30 | 18.91 | 19.15 | 17.97 | 17.18 | 16.86 |  | -0.24 | 1.19 | 0.79 | 0.32 |
| 310 | 30 | 18.83 | 19.10 | 17.87 | 17.08 | 16.78 |  | -0.27 | 1.23 | 0.79 | 0.30 |
| 320 | 30 | 18.83 | 19.11 | 17.86 | 17.06 | 16.78 |  | -0.28 | 1.25 | 0.79 | 0.28 |
| 330 | 30 | 18.81 | 19.19 | 17.91 | 17.12 | 16.82 |  | -0.39 | 1.28 | 0.79 | 0.30 |
| 340 | 30 | 18.95 | 19.32 | 18.05 | 17.26 | 16.93 |  | -0.37 | 1.27 | 0.78 | 0.33 |
| 350 | 30 | 19.13 | 19.47 | 18.17 | 17.39 | 17.00 |  | -0.34 | 1.30 | 0.78 | 0.39 |
| 360 | 30 | 19.41 | 19.69 | 18.44 | 17.67 | 17.24 |  | -0.28 | 1.24 | 0.78 | 0.43 |

**Table S-5** The average NSB in different colors and the color indices over the three observing nights at Alt 40^o^

| Az  Degrees | Alt  Degrees | U_avg_  mag/arcsec2 | B_avg_  mag/arcsec^2^ | V_avg_  mag/arcsec^2^ | R_avg_  mag/arcsec^2^ | I_avg_  mag/arcsec^2^ |  | U-B | B-V | V-R | R-I |
| --- | --- | --- | --- | --- | --- | --- | --- | --- | --- | --- | --- |
|  |  |  |  |  |  |  |  |  |  |  |  |
| 0 | 40 | 19.69 | 19.93 | 18.74 | 17.96 | 17.49 |  | -0.24 | 1.19 | 0.77 | 0.47 |
| 10 | 40 | 19.82 | 20.05 | 18.86 | 18.10 | 17.59 |  | -0.23 | 1.19 | 0.77 | 0.51 |
| 20 | 40 | 19.93 | 20.15 | 18.97 | 18.21 | 17.66 |  | -0.22 | 1.17 | 0.77 | 0.55 |
| 30 | 40 | 20.02 | 20.25 | 19.07 | 18.30 | 17.70 |  | -0.23 | 1.18 | 0.76 | 0.60 |
| 40 | 40 | 20.07 | 20.30 | 19.14 | 18.38 | 17.76 |  | -0.23 | 1.17 | 0.76 | 0.62 |
| 50 | 40 | 20.18 | 20.38 | 19.24 | 18.47 | 17.83 |  | -0.20 | 1.14 | 0.76 | 0.64 |
| 60 | 40 | 20.27 | 20.44 | 19.31 | 18.55 | 17.88 |  | -0.17 | 1.12 | 0.76 | 0.68 |
| 70 | 40 | 20.26 | 20.47 | 19.32 | 18.59 | 17.92 |  | -0.21 | 1.15 | 0.73 | 0.67 |
| 80 | 40 | 20.35 | 20.50 | 19.40 | 18.63 | 17.95 |  | -0.15 | 1.10 | 0.76 | 0.68 |
| 90 | 40 | 20.27 | 20.52 | 19.39 | 18.62 | 17.94 |  | -0.25 | 1.13 | 0.77 | 0.68 |
| 100 | 40 | 20.40 | 20.53 | 19.44 | 18.68 | 17.99 |  | -0.13 | 1.09 | 0.76 | 0.69 |
| 110 | 40 | 20.41 | 20.54 | 19.44 | 18.68 | 17.99 |  | -0.12 | 1.10 | 0.76 | 0.68 |
| 120 | 40 | 20.55 | 20.54 | 19.50 | 18.73 | 18.06 |  | 0.01 | 1.04 | 0.77 | 0.67 |
| 130 | 40 | 20.55 | 20.54 | 19.50 | 18.74 | 18.09 |  | 0.00 | 1.04 | 0.77 | 0.65 |
| 140 | 40 | 20.43 | 20.53 | 19.45 | 18.76 | 18.03 |  | -0.11 | 1.08 | 0.69 | 0.73 |
| 150 | 40 | 20.42 | 20.53 | 19.46 | 18.75 | 18.03 |  | -0.11 | 1.07 | 0.71 | 0.72 |
| 160 | 40 | 20.56 | 20.50 | 19.50 | 18.79 | 18.07 |  | 0.05 | 1.00 | 0.71 | 0.73 |
| 170 | 40 | 20.55 | 20.43 | 19.47 | 18.77 | 18.03 |  | 0.12 | 0.96 | 0.71 | 0.74 |
| 180 | 40 | 20.35 | 20.46 | 19.38 | 18.64 | 17.98 |  | -0.11 | 1.08 | 0.74 | 0.66 |
| 190 | 40 | 20.32 | 20.41 | 19.36 | 18.61 | 17.96 |  | -0.09 | 1.05 | 0.75 | 0.65 |
| 200 | 40 | 20.26 | 20.37 | 19.28 | 18.53 | 17.92 |  | -0.11 | 1.08 | 0.75 | 0.61 |
| 210 | 40 | 20.17 | 20.29 | 19.19 | 18.44 | 17.85 |  | -0.13 | 1.11 | 0.75 | 0.59 |
| 220 | 40 | 20.08 | 20.22 | 19.08 | 18.34 | 17.78 |  | -0.14 | 1.14 | 0.74 | 0.56 |
| 230 | 40 | 19.97 | 20.12 | 18.99 | 18.26 | 17.73 |  | -0.15 | 1.14 | 0.73 | 0.54 |
| 240 | 40 | 19.85 | 20.02 | 18.89 | 18.14 | 17.63 |  | -0.17 | 1.13 | 0.75 | 0.51 |
| 250 | 40 | 19.72 | 19.93 | 18.77 | 18.02 | 17.54 |  | -0.21 | 1.15 | 0.75 | 0.48 |
| 260 | 40 | 19.62 | 19.82 | 18.67 | 17.91 | 17.44 |  | -0.21 | 1.16 | 0.76 | 0.47 |
| 270 | 40 | 19.52 | 19.72 | 18.57 | 17.80 | 17.35 |  | -0.21 | 1.15 | 0.77 | 0.46 |
| 280 | 40 | 19.42 | 19.64 | 18.48 | 17.71 | 17.27 |  | -0.22 | 1.17 | 0.76 | 0.45 |
| 290 | 40 | 19.35 | 19.57 | 18.40 | 17.63 | 17.19 |  | -0.22 | 1.17 | 0.77 | 0.44 |
| 300 | 40 | 19.27 | 19.53 | 18.32 | 17.50 | 17.16 |  | -0.27 | 1.21 | 0.82 | 0.35 |
| 310 | 40 | 19.21 | 19.49 | 18.27 | 17.50 | 17.12 |  | -0.27 | 1.22 | 0.77 | 0.38 |
| 320 | 40 | 19.14 | 19.49 | 18.21 | 17.44 | 17.10 |  | -0.35 | 1.28 | 0.77 | 0.34 |
| 330 | 40 | 19.23 | 19.59 | 18.28 | 17.51 | 17.14 |  | -0.36 | 1.31 | 0.77 | 0.37 |
| 340 | 40 | 19.35 | 19.68 | 18.41 | 17.64 | 17.24 |  | -0.33 | 1.27 | 0.77 | 0.40 |
| 350 | 40 | 19.48 | 19.79 | 18.53 | 17.77 | 17.33 |  | -0.30 | 1.26 | 0.76 | 0.44 |
| 360 | 40 | 19.69 | 19.91 | 18.74 | 17.99 | 17.50 |  | -0.22 | 1.17 | 0.75 | 0.49 |

**Table S-6** The average NSB in different colors and the color indices over the three observing nights at Alt 50^o^

| Az  Degrees | Alt  Degrees | U_avg_  mag/arcsec2 | B_avg_  mag/arcsec^2^ | V_avg_  mag/arcsec^2^ | R_avg_  mag/arcsec^2^ | I_avg_  mag/arcsec^2^ |  | U-B | B-V | V-R | R-I |
| --- | --- | --- | --- | --- | --- | --- | --- | --- | --- | --- | --- |
|  |  |  |  |  |  |  |  |  |  |  |  |
| 0 | 50 | 19.89 | 20.11 | 18.96 | 18.21 | 17.69 |  | -0.22 | 1.15 | 0.75 | 0.53 |
| 10 | 50 | 20.00 | 20.21 | 19.06 | 18.31 | 17.75 |  | -0.22 | 1.16 | 0.75 | 0.56 |
| 20 | 50 | 20.08 | 20.28 | 19.14 | 18.39 | 17.79 |  | -0.20 | 1.14 | 0.74 | 0.60 |
| 30 | 50 | 20.09 | 20.33 | 19.18 | 18.42 | 17.81 |  | -0.24 | 1.14 | 0.76 | 0.61 |
| 40 | 50 | 20.21 | 20.38 | 19.26 | 18.52 | 17.88 |  | -0.18 | 1.12 | 0.75 | 0.64 |
| 50 | 50 | 20.30 | 20.43 | 19.34 | 18.58 | 17.93 |  | -0.13 | 1.08 | 0.76 | 0.66 |
| 60 | 50 | 20.33 | 20.48 | 19.40 | 18.65 | 17.98 |  | -0.14 | 1.08 | 0.75 | 0.67 |
| 70 | 50 | 20.38 | 20.51 | 19.44 | 18.70 | 18.02 |  | -0.13 | 1.07 | 0.74 | 0.68 |
| 80 | 50 | 20.41 | 20.54 | 19.47 | 18.72 | 18.03 |  | -0.13 | 1.07 | 0.74 | 0.69 |
| 90 | 50 | 20.43 | 20.55 | 19.48 | 18.73 | 18.05 |  | -0.12 | 1.07 | 0.75 | 0.69 |
| 100 | 50 | 20.45 | 20.56 | 19.50 | 18.76 | 18.07 |  | -0.12 | 1.06 | 0.75 | 0.69 |
| 110 | 50 | 20.45 | 20.57 | 19.51 | 18.76 | 18.07 |  | -0.12 | 1.06 | 0.75 | 0.69 |
| 120 | 50 | 20.45 | 20.57 | 19.52 | 18.76 | 18.08 |  | -0.12 | 1.05 | 0.76 | 0.68 |
| 130 | 50 | 20.44 | 20.58 | 19.50 | 18.75 | 18.07 |  | -0.14 | 1.08 | 0.75 | 0.68 |
| 140 | 50 | 20.46 | 20.58 | 19.52 | 18.77 | 18.10 |  | -0.12 | 1.06 | 0.75 | 0.67 |
| 150 | 50 | 20.47 | 20.57 | 19.52 | 18.78 | 18.10 |  | -0.10 | 1.05 | 0.74 | 0.68 |
| 160 | 50 | 20.46 | 20.55 | 19.50 | 18.80 | 18.09 |  | -0.10 | 1.06 | 0.69 | 0.72 |
| 170 | 50 | 20.45 | 20.52 | 19.49 | 18.75 | 18.05 |  | -0.07 | 1.03 | 0.74 | 0.70 |
| 180 | 50 | 20.55 | 20.49 | 19.51 | 18.81 | 18.14 |  | 0.07 | 0.98 | 0.70 | 0.67 |
| 190 | 50 | 20.54 | 20.47 | 19.48 | 18.79 | 18.11 |  | 0.07 | 0.99 | 0.69 | 0.68 |
| 200 | 50 | 20.49 | 20.42 | 19.43 | 18.73 | 18.07 |  | 0.07 | 0.99 | 0.70 | 0.66 |
| 210 | 50 | 20.44 | 20.38 | 19.36 | 18.67 | 18.02 |  | 0.06 | 1.03 | 0.69 | 0.65 |
| 220 | 50 | 20.36 | 20.33 | 19.27 | 18.58 | 17.95 |  | 0.02 | 1.06 | 0.69 | 0.63 |
| 230 | 50 | 20.29 | 20.26 | 19.22 | 18.54 | 17.93 |  | 0.03 | 1.04 | 0.69 | 0.61 |
| 240 | 50 | 20.21 | 20.19 | 19.13 | 18.44 | 17.85 |  | 0.02 | 1.06 | 0.69 | 0.59 |
| 250 | 50 | 19.89 | 20.11 | 18.99 | 18.29 | 17.72 |  | -0.22 | 1.12 | 0.70 | 0.57 |
| 260 | 50 | 20.01 | 20.05 | 18.98 | 18.27 | 17.72 |  | -0.04 | 1.07 | 0.71 | 0.55 |
| 270 | 50 | 19.93 | 19.98 | 18.91 | 18.19 | 17.66 |  | -0.05 | 1.07 | 0.72 | 0.54 |
| 280 | 50 | 19.87 | 19.92 | 18.84 | 18.12 | 17.60 |  | -0.05 | 1.08 | 0.72 | 0.52 |
| 290 | 50 | 19.80 | 19.89 | 18.77 | 18.06 | 17.53 |  | -0.09 | 1.11 | 0.72 | 0.52 |
| 300 | 50 | 19.74 | 19.84 | 18.72 | 18.01 | 17.51 |  | -0.10 | 1.12 | 0.71 | 0.50 |
| 310 | 50 | 19.72 | 19.81 | 18.69 | 17.94 | 17.40 |  | -0.10 | 1.13 | 0.74 | 0.54 |
| 320 | 50 | 19.53 | 19.84 | 18.58 | 17.84 | 17.39 |  | -0.31 | 1.26 | 0.74 | 0.45 |
| 330 | 50 | 19.60 | 19.88 | 18.67 | 17.92 | 17.46 |  | -0.28 | 1.22 | 0.74 | 0.46 |
| 340 | 50 | 19.68 | 19.95 | 18.75 | 17.99 | 17.52 |  | -0.28 | 1.21 | 0.76 | 0.47 |
| 350 | 50 | 19.79 | 20.01 | 18.83 | 18.09 | 17.58 |  | -0.22 | 1.17 | 0.75 | 0.51 |
| 360 | 50 | 19.90 | 20.12 | 18.97 | 18.23 | 17.68 |  | -0.22 | 1.15 | 0.74 | 0.55 |

**Table S-7** The average NSB in different colors and the color indices over the three observing nights at Alt 60^o^

| Az  Degrees | Alt  Degrees | U_avg_  mag/arcsec^2^ | B_avg_  mag/arcsec^2^ | V_avg_  mag/arcsec^2^ | R_avg_  mag/arcsec^2^ | I_avg_  mag/arcsec^2^ |  | U-B | B-V | V-R | R-I |
| --- | --- | --- | --- | --- | --- | --- | --- | --- | --- | --- | --- |
|  |  |  |  |  |  |  |  |  |  |  |  |
| 0 | 60 | 20.07 | 20.27 | 19.11 | 18.34 | 17.60 |  | -0.20 | 1.16 | 0.77 | 0.74 |
| 10 | 60 | 20.12 | 20.31 | 19.20 | 18.45 | 17.84 |  | -0.19 | 1.11 | 0.75 | 0.62 |
| 20 | 60 | 20.17 | 20.35 | 19.23 | 18.49 | 17.88 |  | -0.18 | 1.12 | 0.74 | 0.62 |
| 30 | 60 | 20.22 | 20.38 | 19.27 | 18.54 | 17.91 |  | -0.16 | 1.10 | 0.74 | 0.63 |
| 40 | 60 | 20.30 | 20.44 | 19.33 | 18.59 | 17.95 |  | -0.13 | 1.11 | 0.74 | 0.64 |
| 50 | 60 | 20.38 | 20.44 | 19.42 | 18.69 | 18.03 |  | -0.05 | 1.02 | 0.73 | 0.66 |
| 60 | 60 | 20.38 | 20.50 | 19.45 | 18.71 | 18.04 |  | -0.12 | 1.06 | 0.74 | 0.66 |
| 70 | 60 | 20.45 | 20.52 | 19.50 | 18.76 | 18.11 |  | -0.06 | 1.02 | 0.74 | 0.65 |
| 80 | 60 | 20.47 | 20.53 | 19.53 | 18.78 | 18.11 |  | -0.06 | 1.00 | 0.75 | 0.67 |
| 90 | 60 | 20.48 | 20.57 | 19.54 | 18.80 | 18.10 |  | -0.08 | 1.02 | 0.74 | 0.70 |
| 100 | 60 | 20.51 | 20.58 | 19.56 | 18.82 | 18.13 |  | -0.07 | 1.02 | 0.74 | 0.69 |
| 110 | 60 | 20.50 | 20.59 | 19.56 | 18.83 | 18.13 |  | -0.09 | 1.03 | 0.73 | 0.70 |
| 120 | 60 | 20.51 | 20.57 | 19.57 | 18.83 | 18.14 |  | -0.05 | 1.00 | 0.74 | 0.69 |
| 130 | 60 | 20.52 | 20.58 | 19.57 | 18.83 | 18.15 |  | -0.06 | 1.01 | 0.74 | 0.68 |
| 140 | 60 | 20.51 | 20.59 | 19.57 | 18.83 | 18.14 |  | -0.08 | 1.02 | 0.74 | 0.69 |
| 150 | 60 | 20.54 | 20.58 | 19.57 | 18.83 | 18.16 |  | -0.03 | 1.01 | 0.74 | 0.67 |
| 160 | 60 | 20.65 | 20.56 | 19.61 | 18.87 | 18.16 |  | 0.09 | 0.95 | 0.74 | 0.71 |
| 170 | 60 | 20.64 | 20.54 | 19.58 | 18.85 | 18.17 |  | 0.10 | 0.96 | 0.73 | 0.68 |
| 180 | 60 | 20.59 | 20.48 | 19.55 | 18.85 | 18.20 |  | 0.11 | 0.92 | 0.70 | 0.65 |
| 190 | 60 | 20.59 | 20.46 | 19.53 | 18.82 | 18.20 |  | 0.13 | 0.93 | 0.71 | 0.62 |
| 200 | 60 | 20.57 | 20.43 | 19.50 | 18.77 | 18.18 |  | 0.14 | 0.93 | 0.73 | 0.59 |
| 210 | 60 | 20.53 | 20.37 | 19.45 | 18.75 | 18.15 |  | 0.15 | 0.92 | 0.71 | 0.60 |
| 220 | 60 | 20.49 | 20.35 | 19.39 | 18.69 | 18.09 |  | 0.13 | 0.96 | 0.70 | 0.60 |
| 230 | 60 | 20.42 | 20.31 | 19.35 | 18.65 | 18.08 |  | 0.11 | 0.96 | 0.70 | 0.57 |
| 240 | 60 | 20.37 | 20.26 | 19.26 | 18.58 | 18.02 |  | 0.10 | 1.01 | 0.67 | 0.56 |
| 250 | 60 | 20.26 | 20.22 | 19.23 | 18.53 | 17.99 |  | 0.05 | 0.98 | 0.70 | 0.54 |
| 260 | 60 | 20.22 | 20.17 | 19.18 | 18.45 | 17.94 |  | 0.05 | 0.99 | 0.73 | 0.52 |
| 270 | 60 | 20.15 | 20.13 | 19.12 | 18.40 | 17.88 |  | 0.02 | 1.00 | 0.72 | 0.52 |
| 280 | 60 | 20.11 | 20.10 | 19.08 | 18.36 | 17.87 |  | 0.01 | 1.02 | 0.72 | 0.50 |
| 290 | 60 | 20.03 | 20.07 | 19.03 | 18.29 | 17.80 |  | -0.04 | 1.04 | 0.74 | 0.49 |
| 300 | 60 | 20.00 | 20.06 | 19.01 | 18.27 | 17.77 |  | -0.07 | 1.05 | 0.74 | 0.50 |
| 310 | 60 | 19.91 | 20.07 | 18.96 | 18.22 | 17.72 |  | -0.16 | 1.11 | 0.74 | 0.50 |
| 320 | 60 | 19.87 | 20.08 | 18.93 | 18.21 | 17.70 |  | -0.21 | 1.15 | 0.73 | 0.51 |
| 330 | 60 | 19.94 | 20.11 | 19.03 | 18.32 | 17.78 |  | -0.17 | 1.08 | 0.72 | 0.54 |
| 340 | 60 | 20.01 | 20.14 | 19.08 | 18.34 | 17.82 |  | -0.14 | 1.06 | 0.74 | 0.52 |
| 350 | 60 | 20.01 | 20.20 | 19.13 | 18.38 | 17.85 |  | -0.18 | 1.07 | 0.74 | 0.53 |
| 360 | 60 | 20.10 | 20.22 | 19.17 | 18.40 | 17.88 |  | -0.12 | 1.05 | 0.77 | 0.52 |

**Table S-8** The average NSB in different colors and the color indices over the three observing nights at Alt 70^o^

| Az  Degrees | Alt  Degrees | U_avg_  mag/arcsec^2^ | B_avg_  mag/arcsec^2^ | V_avg_  mag/arcsec^2^ | R_avg_  mag/arcsec^2^ | I_avg_  mag/arcsec^2^ |  | U-B | B-V | V-R | R-I |
| --- | --- | --- | --- | --- | --- | --- | --- | --- | --- | --- | --- |
|  |  |  |  |  |  |  |  |  |  |  |  |
| 0 | 70 | 20.23 | 20.24 | 19.19 | 18.49 | 17.76 |  | -0.01 | 1.06 | 0.70 | 0.73 |
| 10 | 70 | 20.28 | 20.39 | 19.28 | 18.56 | 17.85 |  | -0.11 | 1.11 | 0.72 | 0.71 |
| 20 | 70 | 20.26 | 20.40 | 19.31 | 18.62 | 17.99 |  | -0.14 | 1.10 | 0.69 | 0.63 |
| 30 | 70 | 20.17 | 20.44 | 19.30 | 18.60 | 18.01 |  | -0.28 | 1.14 | 0.70 | 0.59 |
| 40 | 70 | 20.32 | 20.47 | 19.39 | 18.71 | 18.00 |  | -0.15 | 1.08 | 0.68 | 0.71 |
| 50 | 70 | 20.49 | 20.46 | 19.49 | 18.79 | 18.15 |  | 0.03 | 0.97 | 0.69 | 0.64 |
| 60 | 70 | 20.50 | 20.52 | 19.53 | 18.82 | 18.20 |  | -0.02 | 0.99 | 0.71 | 0.63 |
| 70 | 70 | 20.55 | 20.54 | 19.56 | 18.87 | 18.22 |  | 0.01 | 0.98 | 0.70 | 0.65 |
| 80 | 70 | 20.57 | 20.55 | 19.58 | 18.87 | 18.23 |  | 0.02 | 0.97 | 0.71 | 0.64 |
| 90 | 70 | 20.57 | 20.58 | 19.60 | 18.89 | 18.24 |  | -0.01 | 0.99 | 0.70 | 0.65 |
| 100 | 70 | 20.59 | 20.58 | 19.60 | 18.89 | 18.25 |  | 0.01 | 0.98 | 0.71 | 0.65 |
| 110 | 70 | 20.60 | 20.58 | 19.62 | 18.92 | 18.27 |  | 0.03 | 0.95 | 0.70 | 0.65 |
| 120 | 70 | 20.65 | 20.58 | 19.63 | 18.94 | 18.28 |  | 0.07 | 0.96 | 0.69 | 0.66 |
| 130 | 70 | 20.65 | 20.58 | 19.62 | 18.88 | 18.26 |  | 0.07 | 0.95 | 0.74 | 0.62 |
| 140 | 70 | 20.66 | 20.56 | 19.64 | 18.90 | 18.29 |  | 0.09 | 0.93 | 0.74 | 0.61 |
| 150 | 70 | 20.68 | 20.56 | 19.64 | 18.91 | 18.28 |  | 0.12 | 0.92 | 0.73 | 0.63 |
| 160 | 70 | 20.67 | 20.54 | 19.63 | 18.93 | 18.28 |  | 0.13 | 0.91 | 0.70 | 0.65 |
| 170 | 70 | 20.53 | 20.62 | 19.58 | 18.88 | 18.22 |  | -0.08 | 1.04 | 0.70 | 0.66 |
| 180 | 70 | 20.51 | 20.46 | 19.57 | 18.86 | 18.20 |  | 0.05 | 0.90 | 0.70 | 0.66 |
| 190 | 70 | 20.49 | 20.30 | 19.52 | 18.82 | 18.05 |  | 0.19 | 0.78 | 0.70 | 0.77 |
| 200 | 70 | 20.49 | 20.42 | 19.52 | 18.82 | 18.12 |  | 0.08 | 0.90 | 0.70 | 0.70 |
| 210 | 70 | 20.46 | 20.42 | 19.49 | 18.78 | 18.11 |  | 0.04 | 0.93 | 0.70 | 0.67 |
| 220 | 70 | 20.45 | 20.41 | 19.45 | 18.76 | 18.09 |  | 0.04 | 0.96 | 0.69 | 0.66 |
| 230 | 70 | 20.41 | 20.37 | 19.40 | 18.71 | 18.04 |  | 0.04 | 0.97 | 0.69 | 0.67 |
| 240 | 70 | 20.38 | 20.33 | 19.39 | 18.69 | 18.06 |  | 0.05 | 0.94 | 0.70 | 0.63 |
| 250 | 70 | 20.34 | 20.31 | 19.35 | 18.65 | 18.01 |  | 0.03 | 0.96 | 0.69 | 0.64 |
| 260 | 70 | 20.31 | 20.09 | 19.28 | 18.59 | 17.93 |  | 0.22 | 0.81 | 0.70 | 0.66 |
| 270 | 70 | 20.27 | 20.27 | 19.19 | 18.49 | 17.65 |  | 0.00 | 1.08 | 0.70 | 0.84 |
| 280 | 70 | 20.20 | 20.24 | 19.22 | 18.52 | 17.92 |  | -0.04 | 1.02 | 0.69 | 0.60 |
| 290 | 70 | 20.13 | 20.24 | 19.19 | 18.49 | 17.89 |  | -0.11 | 1.04 | 0.70 | 0.60 |
| 300 | 70 | 19.66 | 20.24 | 19.03 | 18.33 | 17.74 |  | -0.58 | 1.21 | 0.70 | 0.59 |
| 310 | 70 | 20.10 | 20.23 | 19.17 | 18.46 | 17.87 |  | -0.13 | 1.07 | 0.71 | 0.60 |
| 320 | 70 | 20.14 | 20.25 | 19.20 | 18.48 | 17.89 |  | -0.11 | 1.05 | 0.71 | 0.59 |
| 330 | 70 | 20.14 | 20.25 | 19.20 | 18.49 | 17.90 |  | -0.12 | 1.06 | 0.71 | 0.59 |
| 340 | 70 | 20.20 | 20.29 | 19.24 | 18.52 | 17.91 |  | -0.10 | 1.06 | 0.72 | 0.61 |
| 350 | 70 | 20.23 | 20.32 | 19.27 | 18.54 | 17.94 |  | -0.09 | 1.05 | 0.73 | 0.60 |
| 360 | 70 | 20.29 | 20.24 | 19.21 | 18.48 | 17.76 |  | 0.05 | 1.03 | 0.73 | 0.73 |

**Table S-9** The average NSB in different colors and the color indices over the three observing nights at Alt 80^o^

| Az  Degrees | Alt  Degrees | U_avg_  mag/arcsec^2^ | B_avg_  mag/arcsec^2^ | V_avg_  mag/arcsec^2^ | R_avg_  mag/arcsec^2^ | I_avg_  mag/arcsec^2^ |  | U-B | B-V | V-R | R-I |
| --- | --- | --- | --- | --- | --- | --- | --- | --- | --- | --- | --- |
|  |  |  |  |  |  |  |  |  |  |  |  |
| 0 | 80 | 20.44 | 20.38 | 19.31 | 18.56 | 17.60 |  | 0.06 | 1.08 | 0.74 | 0.97 |
| 10 | 80 | 20.31 | 20.30 | 19.39 | 18.60 | 17.83 |  | 0.01 | 0.92 | 0.78 | 0.77 |
| 20 | 80 | 20.53 | 20.30 | 19.01 | 18.14 | 17.04 |  | 0.23 | 1.29 | 0.87 | 1.09 |
| 30 | 80 | 20.52 | 20.41 | 19.43 | 18.74 | 17.94 |  | 0.11 | 0.99 | 0.69 | 0.80 |
| 40 | 80 | 20.55 | 20.34 | 19.48 | 18.78 | 18.10 |  | 0.21 | 0.86 | 0.70 | 0.68 |
| 50 | 80 | 20.50 | 20.40 | 19.39 | 18.64 | 17.89 |  | 0.11 | 1.00 | 0.76 | 0.75 |
| 60 | 80 | 20.54 | 20.46 | 19.49 | 18.79 | 18.04 |  | 0.08 | 0.97 | 0.70 | 0.75 |
| 70 | 80 | 20.48 | 20.48 | 19.54 | 18.83 | 18.13 |  | 0.01 | 0.94 | 0.71 | 0.70 |
| 80 | 80 | 20.60 | 20.50 | 19.59 | 18.88 | 18.20 |  | 0.10 | 0.91 | 0.71 | 0.68 |
| 90 | 80 | 20.61 | 20.51 | 19.59 | 18.90 | 18.20 |  | 0.10 | 0.91 | 0.70 | 0.69 |
| 100 | 80 | 20.64 | 20.52 | 19.61 | 18.91 | 18.22 |  | 0.11 | 0.91 | 0.70 | 0.69 |
| 110 | 80 | 20.66 | 20.53 | 19.62 | 18.92 | 18.22 |  | 0.13 | 0.92 | 0.70 | 0.70 |
| 120 | 80 | 20.66 | 20.53 | 19.62 | 18.92 | 18.23 |  | 0.12 | 0.91 | 0.70 | 0.69 |
| 130 | 80 | 20.66 | 20.53 | 19.62 | 18.92 | 18.23 |  | 0.13 | 0.91 | 0.70 | 0.69 |
| 140 | 80 | 20.67 | 20.52 | 19.63 | 18.92 | 18.24 |  | 0.15 | 0.89 | 0.70 | 0.68 |
| 150 | 80 | 20.66 | 20.53 | 19.62 | 18.92 | 18.23 |  | 0.13 | 0.91 | 0.70 | 0.69 |
| 160 | 80 | 20.63 | 20.51 | 19.61 | 18.91 | 18.23 |  | 0.12 | 0.90 | 0.70 | 0.68 |
| 170 | 80 | 20.61 | 20.52 | 19.58 | 18.88 | 18.19 |  | 0.09 | 0.93 | 0.70 | 0.69 |
| 180 | 80 | 20.49 | 20.46 | 19.37 | 18.63 | 17.58 |  | 0.03 | 1.09 | 0.74 | 1.05 |
| 190 | 80 | 20.46 | 20.49 | 19.44 | 18.72 | 17.87 |  | -0.03 | 1.05 | 0.72 | 0.86 |
| 200 | 80 | 20.57 | 20.45 | 19.50 | 18.78 | 18.07 |  | 0.12 | 0.95 | 0.72 | 0.71 |
| 210 | 80 | 20.60 | 20.49 | 19.53 | 18.80 | 18.14 |  | 0.11 | 0.96 | 0.72 | 0.66 |
| 220 | 80 | 20.58 | 20.42 | 19.52 | 18.80 | 18.16 |  | 0.16 | 0.90 | 0.72 | 0.65 |
| 230 | 80 | 20.54 | 20.16 | 19.49 | 18.77 | 18.12 |  | 0.38 | 0.67 | 0.72 | 0.65 |
| 240 | 80 | 20.54 | 20.11 | 19.47 | 18.61 | 18.08 |  | 0.43 | 0.64 | 0.86 | 0.53 |
| 250 | 80 | 20.04 | 19.91 | 19.07 | 18.30 | 17.20 |  | 0.12 | 0.85 | 0.76 | 1.10 |
| 260 | 80 | 20.36 | 20.37 | 18.98 | 18.16 | 17.01 |  | -0.02 | 1.40 | 0.82 | 1.15 |
| 270 | 80 | 19.80 | 20.17 | 19.02 | 18.29 | 17.55 |  | -0.37 | 1.15 | 0.73 | 0.74 |
| 280 | 80 | 20.33 | 20.02 | 19.00 | 18.26 | 17.47 |  | 0.31 | 1.02 | 0.74 | 0.78 |
| 290 | 80 | 20.43 | 20.39 | 19.30 | 18.58 | 17.97 |  | 0.04 | 1.09 | 0.72 | 0.60 |
| 300 | 80 | 20.39 | 20.41 | 19.35 | 18.63 | 18.04 |  | -0.02 | 1.06 | 0.72 | 0.59 |
| 310 | 80 | 20.44 | 20.41 | 19.36 | 18.65 | 18.03 |  | 0.03 | 1.05 | 0.71 | 0.62 |
| 320 | 80 | 20.46 | 20.41 | 19.36 | 18.65 | 18.04 |  | 0.06 | 1.04 | 0.71 | 0.62 |
| 330 | 80 | 20.45 | 20.39 | 19.36 | 18.67 | 18.05 |  | 0.06 | 1.03 | 0.69 | 0.62 |
| 340 | 80 | 20.47 | 20.44 | 19.39 | 18.67 | 18.08 |  | 0.04 | 1.04 | 0.73 | 0.59 |
| 350 | 80 | 20.48 | 20.38 | 19.44 | 18.73 | 18.10 |  | 0.09 | 0.95 | 0.71 | 0.63 |
| 360 | 80 | 20.47 | 20.44 | 19.40 | 18.53 | 17.51 |  | 0.04 | 1.04 | 0.87 | 1.02 |

**Table S-10** The average NSB in different colors and the color indices over the three observing nights at Alt 90^o^

| Az  Degrees | Alt  Degrees | U_avg_  mag/arcsec^2^ | B_avg_  mag/arcsec^2^ | V_avg_  mag/arcsec^2^ | R_avg_  mag/arcsec^2^ | I_avg_  mag/arcsec^2^ |  | U-B | B-V | V-R | R-I |
| --- | --- | --- | --- | --- | --- | --- | --- | --- | --- | --- | --- |
|  |  |  |  |  |  |  |  |  |  |  |  |
| 0 | 90 | 20.44 | 20.26 | 19.27 | 18.57 | 17.84 |  | 0.18 | 0.99 | 0.70 | 0.73 |
| 10 | 90 | 20.29 | 20.44 | 19.38 | 18.68 | 17.93 |  | -0.15 | 1.06 | 0.70 | 0.75 |
| 20 | 90 | 20.24 | 20.47 | 19.37 | 18.67 | 18.02 |  | -0.23 | 1.10 | 0.70 | 0.65 |
| 30 | 90 | 20.46 | 20.47 | 19.48 | 18.70 | 17.68 |  | -0.01 | 0.99 | 0.78 | 1.01 |
| 40 | 90 | 20.44 | 20.51 | 19.47 | 18.78 | 17.98 |  | -0.06 | 1.04 | 0.69 | 0.79 |
| 50 | 90 | 20.53 | 20.03 | 19.47 | 18.77 | 18.11 |  | 0.49 | 0.57 | 0.70 | 0.66 |
| 60 | 90 | 20.55 | 20.08 | 19.41 | 18.70 | 17.99 |  | 0.46 | 0.68 | 0.71 | 0.71 |
| 70 | 90 | 20.26 | 20.28 | 18.36 | 17.55 | 17.89 |  | -0.02 | 1.92 | 0.81 | -0.34 |
| 80 | 90 | 20.45 | 20.40 | 19.38 | 18.27 | 17.30 |  | 0.05 | 1.02 | 1.12 | 0.97 |
| 90 | 90 | 20.52 | 20.50 | 19.04 | 18.29 | 17.45 |  | 0.01 | 1.46 | 0.75 | 0.84 |
| 100 | 90 | 20.58 | 20.54 | 19.53 | 18.82 | 18.15 |  | 0.04 | 1.01 | 0.71 | 0.67 |
| 110 | 90 | 20.59 | 20.54 | 19.53 | 18.84 | 18.17 |  | 0.05 | 1.01 | 0.70 | 0.67 |
| 120 | 90 | 20.60 | 20.54 | 19.54 | 18.82 | 18.18 |  | 0.06 | 1.00 | 0.72 | 0.64 |
| 130 | 90 | 20.59 | 20.54 | 19.53 | 18.80 | 18.18 |  | 0.06 | 1.00 | 0.73 | 0.62 |
| 140 | 90 | 20.61 | 20.54 | 19.53 | 18.80 | 18.16 |  | 0.07 | 1.01 | 0.73 | 0.63 |
| 150 | 90 | 20.59 | 20.54 | 19.53 | 18.80 | 18.15 |  | 0.05 | 1.01 | 0.74 | 0.64 |
| 160 | 90 | 20.61 | 20.53 | 19.54 | 18.80 | 18.16 |  | 0.07 | 1.00 | 0.74 | 0.64 |
| 170 | 90 | 20.59 | 20.54 | 19.55 | 18.80 | 18.17 |  | 0.04 | 1.00 | 0.75 | 0.63 |
| 180 | 90 | 20.49 | 20.43 | 19.43 | 18.68 | 17.97 |  | 0.06 | 1.00 | 0.75 | 0.71 |
| 190 | 90 | 20.56 | 20.47 | 19.45 | 18.70 | 17.97 |  | 0.09 | 1.02 | 0.75 | 0.72 |
| 200 | 90 | 20.58 | 20.48 | 19.47 | 18.71 | 18.03 |  | 0.09 | 1.01 | 0.76 | 0.68 |
| 210 | 90 | 20.57 | 20.52 | 19.52 | 18.75 | 18.09 |  | 0.05 | 1.00 | 0.77 | 0.65 |
| 220 | 90 | 20.53 | 20.54 | 19.48 | 18.74 | 18.06 |  | -0.01 | 1.06 | 0.74 | 0.67 |
| 230 | 90 | 20.59 | 19.24 | 19.54 | 18.79 | 18.14 |  | 1.35 | -0.30 | 0.75 | 0.64 |
| 240 | 90 | 20.59 | 19.03 | 19.54 | 18.78 | 18.17 |  | 1.56 | -0.51 | 0.76 | 0.62 |
| 250 | 90 | 20.00 | 20.42 | 19.25 | 17.46 | 17.87 |  | -0.41 | 1.16 | 1.79 | -0.41 |
| 260 | 90 | 20.05 | 20.37 | 19.18 | 17.40 | 16.41 |  | -0.32 | 1.19 | 1.78 | 0.99 |
| 270 | 90 | 20.44 | 20.39 | 18.92 | 17.58 | 16.54 |  | 0.05 | 1.47 | 1.34 | 1.05 |
| 280 | 90 | 20.51 | 20.52 | 19.49 | 18.79 | 18.13 |  | -0.02 | 1.03 | 0.70 | 0.65 |
| 290 | 90 | 20.49 | 20.52 | 19.49 | 18.79 | 18.13 |  | -0.03 | 1.03 | 0.70 | 0.66 |
| 300 | 90 | 20.50 | 20.52 | 19.49 | 18.79 | 18.14 |  | -0.01 | 1.03 | 0.70 | 0.65 |
| 310 | 90 | 20.53 | 20.51 | 19.49 | 18.80 | 18.14 |  | 0.02 | 1.02 | 0.70 | 0.66 |
| 320 | 90 | 20.54 | 20.51 | 19.50 | 18.80 | 18.15 |  | 0.03 | 1.01 | 0.70 | 0.65 |
| 330 | 90 | 20.57 | 20.51 | 19.52 | 18.82 | 18.15 |  | 0.07 | 0.99 | 0.70 | 0.66 |
| 340 | 90 | 20.53 | 20.51 | 19.51 | 18.80 | 18.14 |  | 0.02 | 1.01 | 0.70 | 0.66 |
| 350 | 90 | 20.59 | 20.52 | 19.52 | 18.82 | 18.16 |  | 0.07 | 1.00 | 0.70 | 0.66 |
| 360 | 90 | 20.52 | 20.29 | 19.30 | 18.60 | 17.88 |  | 0.22 | 1.00 | 0.70 | 0.71 |

**Fig. S-1** The variation of the average sky brightness in the UBVRI with different azimuth degrees at Alt 0°

**Fig. S-2** The variation of the average sky brightness in the UBVRI with different azimuth degrees at Alt 30°

**Fig. S-3** The variation of the average sky brightness in the UBVRI with different azimuth degrees at Alt 60°

**Fig. S-4** The variation of the average sky brightness in the UBVRI with different azimuth degrees at Alt 90°
